# Absence of SARS-CoV-2 Transmission from Children in Isolation to Guardians, South Korea 

**DOI:** 10.3201/eid2701.203450

**Published:** 2021-01

**Authors:** Eun Joo Lee, Dong Hyun Kim, Sung Hee Chang, Sun Bok Suh, Jina Lee, Hyunju Lee, Mi Seon Han

**Affiliations:** Seongnam Citizens Medical Center, Seongnam, South Korea (E.J. Lee);; Inha University Hospital, Incheon, South Korea (D.H. Kim);; Seoul Medical Center, Seoul, South Korea (S.H. Chang);; Busan Medical Center, Busan, South Korea (S.B. Suh);; Asan Medical Center, University of Ulsan College of Medicine, Seoul (J. Lee);; Seoul National University Bundang Hospital, Seoul National University College of Medicine, Seongnam (H. Lee);; Seoul Metropolitan Government-Seoul National University Boramae Medical Center, Seoul (M.S. Han)

**Keywords:** respiratory infections, severe acute respiratory syndrome coronavirus 2, SARS-CoV-2, SARS, COVID-19, coronavirus disease, zoonoses, viruses, coronavirus, children, South Korea

## Abstract

We explored transmission of severe acute respiratory syndrome coronavirus 2 among 12 children and their uninfected guardians in hospital isolation rooms in South Korea. We found that, even with close frequent contact, guardians who used appropriate personal protective equipment were not infected by children with diagnosed coronavirus disease.

Coronavirus disease (COVID-19) in children is known to occur mainly from family clusters (*1*). However, children can be the only infected members in a household, especially when COVID-19 is contracted from relatives or teachers. Such situations raise concerns about isolation because little information is available on transmission of severe acute respiratory syndrome coronavirus 2 (SARS-CoV-2), which causes COVID-19, during colocation of young children with their uninfected guardians. Although children generally are asymptomatic or have mild symptoms, they could be infective (*1*,*2*). We explored whether SARS-CoV-2 was transmitted from children to their uninfected guardians in a hospital isolation setting.

During February 18–June 7, 2020, we analyzed all children <19 years of age with COVID-19 and their uninfected guardians who were isolated together in 7 hospitals in South Korea. The infected children were encouraged to wear face masks. The guardians were advised to wear personal protective equipment (PPE), but the degree of PPE varied among hospitals. Adherence to PPE was monitored by the medical staff; compliance was judged as good when PPE was worn most of the time, fair for frequent adherence, and poor when PPE was worn for less than half of the observed time. Children’s isolation was lifted when 2 consecutive negative respiratory samples were obtained >24 hours apart. To ascertain secondary transmission, guardians’ respiratory samples were tested for SARS-CoV-2 if symptoms developed, before their child’s isolation was lifted, and 2 weeks after the end of isolation. This study was approved by the institutional review board of each hospital and written informed consent was waived.

Among 94 children with COVID-19 isolated in 7 hospitals, 12 children were isolated with a single uninfected guardian (Table). The median age of the patients was 6 years (range 2 months–11 years), and children were isolated for a median of 17 days (range 7–37 days). Most (7/12) children were asymptomatic, 4 had fever or respiratory symptoms, and 1 had pneumonia. Only 4 children cooperated well with wearing face masks.

**Table:** Clinical characteristics and infection control measures of 12 children with COVID-19 and their uninfected guardians sharing hospital isolation rooms, South Korea, February 18–June 7, 2020*

Characteristics	Patient no.											
	1	2	3	4	5	6	7	8	9	10	11	12
Patient sex	F	F	M	F	M	M	M	M	F	M	M	M
Patient age, y	6	9	8	9	11	8	0	6	6	5	4	4
Days of isolation	7	37	17	9	15	21	19	12	16	31	30	17
Symptoms	None	None	Mild FV, C	None	None	None	FV, C, S	None	None	FV, ST	FV, V	C, S
Pneumonia	N	N	Y	N	N	N	N	N	N	N	N	N
Face mask†	KF94	Surgical	Surgical	Surgical	Surgical	Surgical	None	KF94	KF94	KF80	None	KF94
Compliance	Good	Good	Poor	Good	Good	Poor	NA	Fair	Fair	Fair	NA	Poor
Guardian	Mother	Mother	Mother	Mother	Mother	Mother	Father	Mother	Uncle	Mother	Mother	Mother
Symptoms‡	None	None	None	None	None	None	None	None	None	None	None	C, S, H
Face mask	KF94	KF94	N95	N95	N95	N95	KF94	KF94	KF94	KF80	Surgical	KF94
Goggles	N	Y	Y	Y	Y	Y	Y	N	N	N	N	N
Gloves	Y	Y	Y	Y	Y	Y	Y	Y	Y	Y	N	Y
Gown/coverall	Y/N	Y/N	N/Y	N/Y	N/Y	N/Y	N/Y	N/N	N/N	N/N	N/N	Y/N
Contact with patient§	>1 m	Close	Close	Close	Close	Close	Close	Close	>1 m	Close	Close	Close

*Guardians were with the children for the duration of their isolation. COVID-19, coronavirus disease; C, cough; FV, fever; H, headache; NA, not applicable; S, sputum; ST, sore throat; V, vomiting.  †Compliance was deemed good when masks were worn most of the time, fair for frequent adherence, and poor when worn <50% of observed time. A KF94 mask filters ≈94% of particles of 0.4 μm in size, an N95 mask filters ≈95% of particles of 0.3 μm in size, and a KF80 mask filters ≈80% of particles of 0.6 μm. ‡None of the guardians tested positive for COVID-19. §Guardians for patients 1 and 9 maintained >1-meter distance from patients, but other guardians had frequent close contact with patients.

The guardians included 10 mothers, 1 father, and 1 uncle; all complied with wearing PPE (Table). Most (10/12) guardians wore gloves and masks, either KF94 masks, which filter »94% of particles of 0.4 μm in size, or N95 masks which filter »95% of particles of 0.3 μm in size; 7 also wore gowns or coveralls. One guardian used a surgical mask and 1 guardian wore a KF80 mask, which filters »80% of particles of 0.6 μm, and gloves. Most (10/12) guardians had frequent close contact, but 2 children kept a distance of >1 m from their guardians during isolation. None of the guardians were SARS-CoV-2–positive during the study.

For comparison, we also analyzed 2 cases in which adults with COVID-19 were isolated with their uninfected children because no other caregivers were available (Appendix). The adult patients always wore face masks, but the 2 children never wore PPE and always had physical contact with their parents. However, the children did not become infected. The infected parents’ adherence to the use of masks likely aided in curbing SARS-CoV-2 transmission to their uninfected children by reducing virus particles in respiratory droplets (*3*).

Appropriate use of PPE, especially face masks, might have protected uninfected guardians in our study. Previous reports have emphasized the use of face masks to prevent SARS-CoV-2 transmission in healthcare and community settings (*4*). Considering the decreased risk for virus transmission noted with PPE, guardians should be counseled on the proper use of PPE when in isolation with infected children.

We did not observe SARS-CoV-2 transmission from children to guardians in isolation settings in which close proximity would seem to increase transmission risk. Recent studies have suggested that children are not the main drivers of the COVID-19 pandemic, although the reasons remain unclear (*5*). A large study on contacts of COVID-19 case-patients in South Korea observed that household transmission was lowest when the index case-patient was 0–9 years of age (*6*). Among pediatric cases, the secondary attack rate from children to household members was estimated to be only 0.5% (*7*). Reduced transmission from children in households was also reported in Switzerland and China and in educational settings in Australia (*8*–*10*). 

This study is limited by its small sample size, which limits the ability to generalize its results. Moreover, we did not assess the patients’ viral load, which could indirectly reflect the infectivity of the children, nor did we assess patient serology, which could further ascertain their infection status. Despite these limitations, our study provides information on SARS-CoV-2 transmission from children to guardians in isolation rooms. Additional assessments of the transmissibility of SARS-CoV-2 by children and the role of PPE in preventing infection could provide guidance during the ongoing pandemic. Nonetheless, our study adds to growing evidence that young children are less likely to contribute to the spread of COVID-19 among their adult guardians. 

Appendix Additional characteristics and infection control measures for 2 adults with coronavirus disease and their uninfected children in isolation rooms, South Korea.
