# Supplementary material for: Absence of SARS-CoV-2 Transmission from Children in Isolation to Guardians, South Korea
Source: Emerg Infect Dis. 2021 Jan;27(1):308–10. doi: 10.3201/eid2701.203450 (PMC7774560; doi:10.3201/eid2701.203450)
Supplement: Appendix — Additional characteristics and infection control measures for 2 adults with coronavirus disease and their uninfected children in isolation rooms, South Korea. [file 20-3450-Techapp-s1.pdf]

Article DOI: https://doi.org/10.3201/eid2701.203450

Absence of SARS-CoV-2 Transmission from Children in Isolation to Guardians, South Korea

Appendix

Appendix Table. Clinical characteristics and infection control measures of 2 adults with COVID-19 and their uninfected children who shared isolation rooms, South Korea, February 18–June 7, 2020*

Characteristics	Adult patient 1	Adult patient 2
Sex	F	M
Age, y	33	41
Days of isolation	25	20
Symptoms	Anosmia	Fever, sore throat, myalgia, cough, sputum, rhinorrhea
Pneumonia	No	Yes
Face mask†	KF94	Surgical
Compliance	Good	Good
Child	Son	Son
Age, mo.	23	17
Contact with patient	Frequent close contact	Frequent close contact
Symptoms‡	Mild fever	Cough, rhinorrhea
Face mask	–	–
Goggles	–	–
Gloves	–	–
Gown/coverall	−/−	−/−

*COVID-19, coronavirus disease; –, not used.
†A KF94 mask filters ≈94% of particles of 0.4 µm in size.
‡Neither child tested positive for COVID-19.
